# Glutathione and neurodegenerative diseases: immunopharmacological implications

**DOI:** 10.3389/fphar.2025.1737199

**Published:** 2026-01-15

**Authors:** Ethan Knudsen, Jaxson Tadje, Carter Coggins, Vishwanath Venketaraman

**Affiliations:** Department of Microbiology and Immunology, College of Osteopathic Medicine of the Pacific, Western University of Health Sciences, Pomona, CA, United States

**Keywords:** Alzheimer’s disease, glutathione, multiple sclerosis, Parkinson’s disease, redox imbalance

## Abstract

Neurodegenerative diseases are characterized by progressive neuronal dysfunction, often accompanied by chronic inflammatory states and redox imbalance within the central nervous system (CNS). Glutathione (GSH), a key regulator of oxidative stress and cellular immunity, has a critical role in modulating the functional states of CNS-resident and infiltrating immune cell subsets. This review aims to synthesize emerging evidence on how GSH depletion contributes to impaired immune and antioxidant activity in neurodegenerative diseases, such as Parkinson’s Disease (PD), Alzheimer’s Disease (AD), and multiple sclerosis (MS). By exploring how redox signaling via GSH influences inflammatory immune phenotypes across different disease states, we will isolate possible therapeutic interventions for treatment of these conditions. By characterizing GSH’s function and designating it as a special regulator of immune cell behavior, this review highlights its potential as both a therapeutic agent and biomarker for patients with neurodegenerative conditions.

## 1 Introduction

Neurodegenerative diseases such as Alzheimer’s disease (AD), Parkinson’s disease (PD), and multiple sclerosis (MS) are characterized by progressive nerve damage and chronic inflammation. While these diseases have distinct etiologies and pathologies, they all involve neuronal loss and sustained activation of the brain’s immune system, including microglia and astrocytes (Ferreira et al., 2013). This neuroinflammatory response not only contributes to disease progression, but also amplifies damage initiated by other pathological processes, such as protein aggregation, mitochondrial dysfunction, and synaptic failure.

A key driver of neuronal injury is oxidative stress, which arises when the generation of reactive oxygen species (ROS) overwhelms the brain’s antioxidant defenses, one such defense being glutathione (GSH) (Dringen and Arend, 2025). Neurons are highly vulnerable to oxidative damage due to their high metabolic rate and limited capacity for regeneration (Cobley et al., 2018). ROS-induced damage to DNA, proteins, and lipids can directly lead to cell death, but it also promotes microglial activation and the release of pro-inflammatory cytokines, further exacerbating neurodegeneration (Tfilin et al., 2023). Oxidative stress and neuroinflammation create a reciprocal pathological process that facilitates redox imbalance and contributes to immune dysregulation in the central nervous system (CNS).

GSH is the most abundant endogenous antioxidant in the brain and plays an important role in counteracting oxidative stress (Aoyama, 2021). A tripeptide composed of glutamate, cysteine, and glycine, GSH acts as a redox buffer by detoxifying ROS and maintaining protein thiol homeostasis. In addition to its antioxidant activity, GSH has a variety of other functions including regulation of mitochondrial function, iron metabolism, immune cell polarization, cytokine production, and inflammatory signaling (Lu, 2013; Kwon et al., 2019). In the CNS, GSH is synthesized primarily by astrocytes and transported to neurons, where it provides oxidative resistance (Dringen and Arend, 2025). Disruptions to the synthesis, recycling, or cellular distribution of GSH are prominent observations that exist in multiple neurodegenerative diseases, suggesting a possible route of therapeutics that could facilitate treatment of these conditions.

This review focuses specifically on how GSH affects immune cells in the brain, specifically CNS-resident glial cells such as microglia and astrocytes. As an example, GSH depletion has been shown to promote pro-inflammatory microglial phenotypes and amplify astrocyte-microglia crosstalk (Vilhardt et al., 2017). Loss of neuronal GSH has also been linked to microglial activation and complement-mediated neurodegeneration, further emphasizing the link between redox imbalance and immune dysregulation (Hashimoto et al., 2023). These findings highlight GSH as a multi-functional molecule that modulates both redox balance and immune cells in the CNS.

Given these roles, GSH has emerged as a promising therapy and biomarker in neurodegenerative diseases. In PD, early GSH depletion in the *substantia nigra* precedes dopaminergic neuron loss, suggesting that redox dysfunction may be an inciting event that leads to eventual neuronal damage (Smeyne and Smeyne, 2013). In AD, altered GSH levels in both the brain and blood have been associated closely with disease progression and cognitive decline (Mandal et al., 2022). In MS, GSH metabolism is impaired in regions of the CNS that have been demyelinated and correlates with mitochondrial dysfunction and axonal injury (Ferreira et al., 2013). Given this information, emerging therapies which target GSH homeostasis such as N-acetylcysteine (NAC), GSH esters, or activation of antioxidant response elements may provide new avenues to interrupt oxidant-driven inflammation in these diseases.

## 2 Mechanisms of redox regulation in the CNS

### 2.1 GSH as an antioxidant in the brain

The brain, being one of the most metabolically active organs in the body, requires a large share of oxygen relative to its mass. Due to this high aerobic demand, the generation of high amounts of ROS and other oxidative by-products is unavoidable (Halliwell, 2006). Neurons and glial cells operate in a constant state of managing oxidative products, therefore making the CNS inherently vulnerable to redox dysregulation. A key molecular buffer against this oxidative state is GSH. The GSH system is the central antioxidant defense system in mammalian cells, including neurons and glia. At its core, this system relies on the reduced form of GSH to directly detoxify ROS and maintain redox balance (Dringen and Arend, 2025).

GSH is synthesized via two ATP-dependent enzymatic steps catalyzed by glutamate-cysteine ligase (GCL) and glutathione synthetase. Once synthesized, GSH serves as a substrate for GSH peroxidases (GPx), which reduce hydrogen peroxide and lipid peroxides into water and lipid alcohols, respectively (Lu, 2013). This reaction converts the reduced form of glutathione (GSH) into oxidized glutathione (GSSG), which is then reduced back to GSH by glutathione reductase (GR), using electrons from NADPH (Dringen and Arend, 2025). Immunologically GSH serves as a redox buffer, with GSH being the active form that upon neutralizing free radicals becomes converted into GSSG (Vairetti et al., 2021). The ratio of GSH:GSSG is a key indicator of cellular oxidative stress with a high ratio (>100:1) showing a resting/healthy cell and a low ratio (as low as 10:1 or even 1:1) indicating oxidative stress (Zitka et al., 2012). This ratio also modulates cellular immune response; a low ratio decreases IL-12 secretion which favors a Th2 response pattern over Th1 (a condition typically associated with chronic inflammation), while an elevated ratio favors a Th1 response (Brundu et al., 2016; Kwon et al., 2019). This relationship has also shown that GSH depletion impairs dendritic cell mediated T-cell activation, further hindering immune response (Kim et al., 2007).

The glutaredoxin (Grx) system, another key player, operates in close association with the GSH pool and serves primarily to regulate protein thiol redox status through reversible de-glutathionylation. Under oxidative conditions, cysteine residues on proteins can form mixed disulfides with GSH. This is a type of post-translational modification known as S-glutathionylation, which protects proteins from irreversible oxidation, but can disrupt their function. After oxidative stress has passed, Grx enzymes in both the cytosol and mitochondria of cells catalyze the removal of these modifications (Ogata et al., 2021). Grx activity depends on both GSH and GR for regeneration of GSH from GSSG, meaning this system is functionally dependent on the GSH system. Without the enabling effect of the GSH system, Grx activity essentially comes to a halt. In neurons, Grx has a protective effect on redox-sensitive enzymes and ion channels, meaning that its dysfunction is implicated in several neurodegenerative disorders involving protein misfolding and redox signaling dysregulation (Ogata et al., 2021).

The final part of this group is the thioredoxin (Trx) system, another thiol-based redox mechanism that is independent of GSH. This system regulates disulfide bond formation, redox-sensitive transcription factors, and apoptosis. Thioredoxins reduce disulfide bonds in oxidized proteins, converting them back to their functional thiol form (Silva-Adaya et al., 2014). Trx becomes oxidized in the process and is regenerated by thioredoxin reductase using NADPH. The Trx system plays a key role in DNA synthesis, anti-apoptotic signaling, and regulation of redox-sensitive transcription factors. Two of these transcription factors, NF-κB and AP-1, are involved in inflammation, cellular proliferation, and apoptosis (Silva-Adaya et al., 2014; Liu et al., 2017; Karin et al., 1997).

While the GSH, Grx, and Trx systems operate via distinct enzymatic pathways they converge functionally to create a cooperative redox buffering machine. The GSH system ensures a high GSH to GSSG ratio and supplies the reducing equivalents required for both Grx activity and overall detoxification of peroxides. Simultaneously, the Grx system relies directly on GSH to repair glutathionylated proteins. Although biochemically separated from the GSH system, the Trx system shares NADPH as a common reducing cofactor and serves as an alternative pathway for reducing disulfide bonds, making this system especially relevant if the GSH system has been compromised or rendered dysfunctional (Silva-Adaya et al., 2014).

**FIGURE 1:**
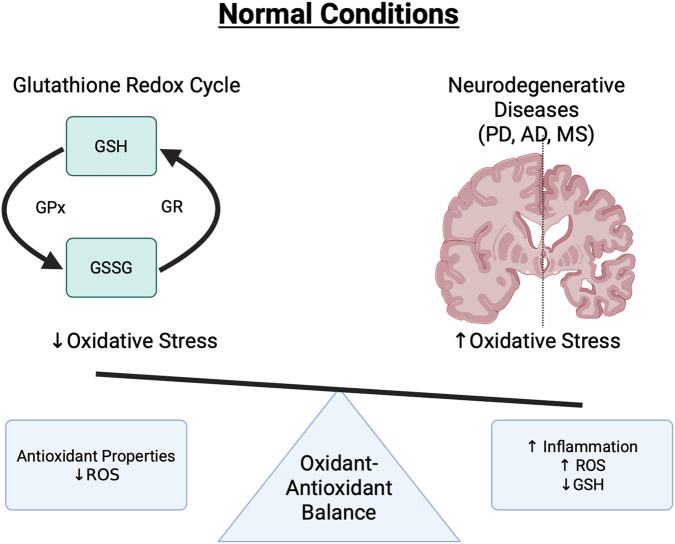
The antioxidant properties of glutathione limit immune attacks in the brain. Reduced glutathione (GSH) inhibits the redox buffering capacity of immune cells leading to a feed-forward loop of inflammation and oxidative stress. This increases ROS, continues to enhance inflammation, and contributes to neurodegenerative disease processes like PD, AD, and MS.

Under oxidative stress these three systems work in concert to detoxify ROS and lipid peroxides, maintain protein structure and function via thiol repair, prevent activation of redox-sensitive inflammatory pathways, and preserve mitochondrial membrane integrity and prevent apoptosis. This special interplay further highlights that the loss of any one system can overwhelm the compensatory capacity of the others, forcing the CNS into a state of redox imbalance, glial activation, and progressive inflammation (Figure 1).

### 2.2 GSH’s role in brain-resident immune cells

In the CNS, GSH protects mitochondrial function, prevents lipid peroxidation, and maintains the redox state of cysteine residues in proteins. Astrocytes are particularly important as they are capable of producing large amounts of GSH and cysteine, both of which are exported to support neurons in the CNS. This mechanism is critical because neurons have a limited ability to synthesize endogenous GS (Dringen and Arend, 2025).

Oxidative stress in the CNS acts as a persistent inflammatory trigger by activating brain-resident immune cells, particularly microglia and astrocytes. ROS and oxidatively modified macromolecules function as damage-associated molecular patterns (DAMPs), engaging pattern recognition receptors (PRRs) on microglia, including Toll-like receptors (TLRs) and NOD-like receptors (NLRs), to initiate inflammatory cascades (Block et al., 2007). Upon activation, microglia upregulate NADPH oxidase 2 which produces superoxide and inducible nitric oxide synthase, ultimately generating nitric oxide. The combination of ROS and reactive nitrogen species (RNS) leads to further oxidative damage and neuronal dysfunction (Block et al., 2007).

Microglia, the resident immune cells of the CNS, are tightly regulated under physiological conditions to maintain neural homeostasis. However, in the context of oxidative stress and limited GSH availability, microglia can become pathologically overactive which contributes to chronic inflammation and neuronal damage. A key redox-signaling pathway present in microglia is the NF-κB pathway. Under normal conditions, NF-κB is sequestered in the cytoplasm by inhibitory proteins known as IκBs. ROS generated during oxidative stress can activate the IκB kinase (IKK) complex, which phosphorylates IκB proteins and marks them for ubiquitin-mediated degradation. This degradation, in turn, allows NF-κB activity to increase, resulting in increased transcription of pro-inflammatory genes, including TNF-α, IL-1β, IL-6, and inducible nitric oxide synthase (iNOS) (Morgan and Liu, 2011). Microglial NF-κB activation under GSH-depleted conditions creates a pro-inflammatory loop where increased ROS promotes NF-κB activation, which upregulates inflammatory cytokines and further stimulates oxidative stress (Liu et al., 2017; Moyse et al., 2022). Importantly, microglia in this activated state also secrete pro-inflammatory cytokines such as TNF-α, IL-1β, and IL-6, which further amplify ROS production and reinforce the inflammatory state.

In addition to the NF-κB pathway, the mitogen-activated protein kinase (MAPK) signaling cascade is also an important regulator of inflammatory responses in CNS microglia. ROS activate the MAPK pathway by modulating redox-sensitive kinases and phosphatases. Specifically, ROS can oxidize and inactivate MAPK phosphatases (MKPs), which normally dephosphorylate and deactivate MAPKs. This deactivation of MKPs allows for the unimpeded progression and sustained activation of the pro-inflammatory ERK1/2, JNK, and p38 signaling cascade within the MAPK pathway (Son et al., 2011).

Astrocytes, the major glial support cells in the CNS, are indispensable in the regulation of GSH homeostasis. They possess a high synthetic capacity for GSH due to high expression of (GCL) and cystine/glutamate antiporter (xCT), and can export GSH to neighboring neurons and glia. Through this intracellular coupling, astrocytes supply neurons with cysteine or cysteinyl-glycine derived from exported GSH, thereby supporting neuronal GSH synthesis and resistance to damaging, redox-driven processes (Kwon et al., 2019). In addition to supplying building blocks for GSH synthesis, GSH derived from astrocytes helps to support astrocyte regulation of the immune system. High GSH content within astrocytes protects them from oxidative stress and preserves their ability for glutamate uptake, ion homeostasis, and immune signaling (Takahashi and Mashima, 2022).

When GSH levels are chronically low, immune cells within the CNS shift to a damage-oriented phenotype. Microglia with depleted GSH have an exacerbated ROS/RNS production, elevated pro-inflammatory cytokine release, impaired phagocytic clearance, and increased neurotoxic output (Lee et al., 2010). Astrocytes with low GSH may become less protective over time, releasing more inflammatory mediators and failing to supply an adequate amount of GSH and GSH precursors to neighboring neurons and microglia (Kwon et al., 2019; Matoba et al., 2023). This dual dysfunction of microglia and astrocytes leads to extensive oxidative stress, immune activation, and eventual neuronal degradation.

**FIGURE 2:**
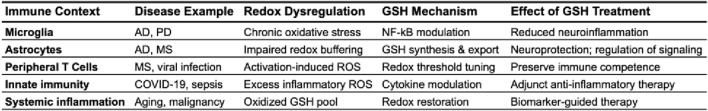
The mechanisms of glutathione treatment and their effects in varying immune contexts.

As discussed, the interplay between astrocytes and microglia via modulation of GSH activity implies that GSH is not only a cellular antioxidant, but an important mediator of cross-talk between resident CNS immune cells. The collapse of both astrocyte and microglia into pro-inflammatory states due to depletion of GSH, leading to sustained neuroinflammation and oxidative stress, is observed in many neurodegenerative diseases (Ferreira et al., 2013; Mandal et al., 2022; Takahashi and Mashima, 2022; Aoyama, 2021). Hence, therapeutic strategies aimed at enhancing glial GSH synthesis or exporting ability may help modulate glial-immune phenotypes, reduce microglial activation, and slow progressive CNS degeneration in neurodegenerative diseases (Figure 2).

## 3 Redox dysregulation in disease states

### 3.1 GSH in peripheral immune cell infiltration

Several studies indicate that neuroinflammatory changes associated with neurodegeneration compromise the BBB or its function by disrupting transport systems, facilitating the infiltration of immune cells, and influencing the BBB’s role as a signaling interface. This multifaceted disruption impairs the BBB’s normal homeostatic roles and directly influences neural activity. Furthermore, recent findings reveal that alterations in BBB and its transporters affect the entry of therapeutic drugs designed to treat neurodegenerative diseases (Carvey et al., 2009). Peripheral immune cells such as T lymphocytes, monocytes, and macrophages thereby infiltrate the CNS and worsen neuroinflammation (Zhang, 2025). As previously stated in this review, GSH is the most abundant intracellular antioxidant and plays major roles in the antioxidant defense system and maintenance of redox homeostasis in both the PNS and CNS (Aoyama K, 2021). With low GSH levels being associated with many chronic pro-inflammatory conditions, such as cardiovascular, renal, and hepatic disease, metabolic syndrome, as well as neurodegenerative conditions and autoimmune disease (Hristov BD, 2022). This is due to the elevated cellular vulnerability to oxidative stress and reactive oxygen species (Gawryluk et al., 2011).

Specifically in regard to T lymphocytes, GSH is necessary for T cell phenotype and effector functions. Normal levels of GSH have been shown to have roles in restricting serine availability to preserve regulatory T cell (Treg) functionality (Kurniawan et al., 2020). Tregs themselves are vital in peripheral immune tolerance and suppressing autoimmunity and inflammation through control of adaptive and innate immune responses (Astarita et al., 2023). In contrast, low GSH skews T cell differentiation toward Th1 and Th17 pro inflammatory states (Johnson et al., 2018). These effector T cells and their cytokines have then been implicated in neuronal injury and glial activation in diseases such as AD and PD through direct cytotoxic effects or through recruitment of immune cells and inflammation (Shi et al., 2022).

Monocytes and macrophages are also influenced by GSH levels. GSH modulates monocyte recruitment and macrophage polarization. High levels of GSH support an anti-inflammatory M2 macrophage phenotype, which is essential for tissue repair and resolving inflammation. In contrast, GSH depletion drives M1 polarization, resulting in heightened secretion of pro-inflammatory cytokines, including TNF-α, IL-6, and IL-1β (Kwon et al., 2019). This is especially damaging in neurodegenerative diseases as the chronic inflammatory state directly contributes to neuronal cell death and synaptic dysfunction. In addition, when GSH is depleted, monocyte-derived macrophages significantly increase their production of reactive oxygen species (ROS), further amplifying oxidative damage within the CNS (Canton et al., 2021). Therefore, these effects are not only local, but the microenvironment that is created maintains M1 activation leading to further, widespread damage.

Overall, GSH helps limit unwanted immune attacks in the brain. Reduced GSH availability, whether due to metabolic dysregulation or chronic ROS exposure impairs the redox buffering capacity of immune cells. Infiltrating immune cells contribute to a feed-forward loop of inflammation and oxidative stress (Fischer and Maier, 2015). This not only enhances their inflammatory potential but also affects antigen processing, glutathionylation of signaling proteins, and interactions with resident CNS cells such as microglia and astrocytes (Federici et al., 2024). In summary, glutathione acts as a critical determinant in regulating the inflammatory potential of infiltrating immune cells.

### 3.2 Alzheimer’s disease

AD is a neurodegenerative condition with gradual onset and progressive impairment of behavioral and cognitive functions. AD is the most common type of dementia, accounting for at least two-thirds of cases in individuals 65 and older (Kumar et al., 2024). Current AD research focuses on understanding pathology by targeting several mechanisms, such as abnormal tau protein metabolism, β-amyloid, inflammatory response, and cholinergic and free radical damage, aiming to develop successful treatments that are capable of stopping or modifying the course of AD (Breijyeh and Karaman, 2020). The two major markers of the disease, β amyloid (Aβ) and tau protein are both closely associated with oxidative stress (Tamagno et al., 2021). GSH is normally highly expressed in microglial cells and aids in microglial phagocytic activity. However, through several magnetic resonance spectroscopy studies (MRS) it has been shown that GSH levels are significantly decreased in the hippocampus, frontal, and cingulate cortices early in AD progression (Mandal et al., 2022). The oxidative stress caused by a decrease in GSH levels can then impact microglia function and potentially lead to a more pro-inflammatory M1 phenotype that has reduced β-amyloid clearance capabilities (Vilhardt et al., 2017). As oxidative stress increases, further stimulation of pro-inflammatory cytokines such as TNF-α and IL-1β increases and contributes to synaptic dysfunction and neuronal degeneration (Dawi et al., 2024).

These studies suggest that the loss of antioxidant defense is an early biochemical event rather than a late-stage consequence of neuronal death. Further studies have provided compelling *in vivo* evidence that GSH levels can serve as a clinically relevant biomarker for mild cognitive impairment (MCI) and AD. In one of these studies, measuring brain GSH levels with *in vivo* proton MRS yielded a correlation in GSH reduction and decline in cognitive functions with 87.5% sensitivity and 100% specificity (Mandal et al., 2015). Another study further tests the correlation between GSH depletion and patients with MCI and results in a similar conclusion (Lin and Lane, 2021). These studies suggest that the loss of antioxidant defense is an early biochemical event rather than a late-stage consequence of neuronal death. Another important correlation between GSH and AD is mitochondrial glutathione (mGSH). MGSH is the main line of defense for the maintenance of the mitochondrial redox environment to avoid and repair oxidative modifications that lead to mitochondrial dysfunction and cell death (Marí et al., 2009). When GSH, and subsequent mGSH, is low, ATP production is impaired. In addition to an increase in mitochondrial cholesterol which further increases β-amyloid, and tau induced toxicity (Ribas et al., 2014). Studies have shown that when amyloid-beta and tau are increased, the cell’s ability to neutralize oxidative stress and mitochondria specific ROS is decreased leading to further tau hyperphosphorylation and neurodegeneration (Alavi Naini and Soussi-Yanicostas 2015). Overall, this simultaneous loss of energy production and insufficient mGSH results in decreased resilience against amyloid-beta and tau, further fueling neuronal loss and leading to increased cell death and an increased rate of neural decline.

These redox imbalances also impact astrocytes, which normally synthesize GSH through the consecutive sections of two ATP consuming cytosolic enzymes. Due to neurons having a limited capacity for GSH synthesis, they must rely on astrocytes to supply precursors such as cysteine (Pérez-Sala D and Pajares, 2023). However, this becomes dysfunctional in AD, further worsening the effects of oxidative stress (Dringen and Arend, 2025). This increased stress as a result of redox imbalance then leaves neurons highly susceptible to the neurotoxic environment.

### 3.3 Parkinson’s disease

PD is a neurodegenerative disorder that typically presents later in life with generalized bradykinesia and another symptom of resting tremor or rigidity. It is estimated that PD affects at least 1% of the population over the age of 60 (Zafar and Yaddanapudi, 2023). The primary pathological hallmark of PD is the progressive loss of dopaminergic neurons in the *substantia nigra pars compacta*. This neurodegeneration is accompanied by the formation of characteristic Lewy bodies, which are intracellular inclusions composed largely of aggregated and misfolded α-synuclein (Balestrino and Schapria, 2020).

This degeneration of dopaminergic neurons is strongly linked to the generation of reactive oxygen species and oxidative stress as well as mitochondrial dysfunction, neuroinflammation, and aging (Dias et al., 2013). One of the earliest biochemical changes seen in PD is a decrease in the levels of total GSH which occurs even before a reduction in mitochondrial complex 1 activity, dopamine levels, or the neurodegeneration that are all associated with the disease (Chinta et al., 2007). Due to the already sensitive nature of dopaminergic neurons to redox imbalance and ROS generation, the additional reduction of an antioxidant like GSH causes neuron loss (Smeyne and Smeyne, 2013). Overall, it has not yet been confirmed that GSH redox imbalance is a causative factor in PD or that PD pathways cause GSH imbalance in PD. However, studies have shown that antioxidant approaches, including anti-neuroinflammatory and neuroprotective agents, may have therapeutic efficacy in the treatment of PD (Balestrino and Schapira, 2020). Suggesting that redox dysfunction is a primary driver that sensitizes neurons to subsequent injury (Bjørklund et al., 2021).

The interplay between mitochondrial function and GSH is especially critical in PD. Specifically, mitochondrial complex I is believed to be the main component of mitochondria affected in PD as Complex I of the electron transport chain is left vulnerable to oxidative damage (Vali et al., 2007). Oxidative stress then accelerates the aggregation of α-synuclein which subsequently binds and sequesters available GSH, creating a destructive feedback loop of protein misfolding and antioxidant loss (Hoepken et al., 2007). This feed-forward mechanism is particularly damaging for dopaminergic neurons as their already high metabolic burden makes them unable to withstand this additional loss of GSH, resulting in apoptosis (Gabby et al., 1996). Even though the definitive role of GSH imbalance in PD is still debated, the efficacy of therapeutic antioxidant and anti-inflammatory agents in PD models underscores the potential of targeting this pathway to halt disease progression (Leathem et al., 2022).

### 3.4 Multiple sclerosis

Multiple sclerosis (MS) is a chronic autoimmune disease that affects the CNS. It is specifically characterized by inflammation, demyelination, and neuronal loss and manifests with a range of neurological symptoms including numbness, tingling, vision impairment, cognitive impairment, and bladder and bowel dysfunction (Tafti et al., 2024). The pathology of MS shows that tissue injury occurs during every stage of disease due to general inflammation as well as focal inflammation that targets the meninges and perivascular spaces. This produces soluble factors which can induce demyelination or neurodegeneration either directly or indirectly through microglia activation (Lassmann, 2018). Additionally, auto reactive effector T cells that penetrate the blood brain barrier are another significant factor in MS. This is critical, as GSH is essential for T cell effector functions through its regulation of metabolic activity (Mak et al., 2017).

In MS, the control of auto-reactive T cells is compromised. Regulatory T (Treg) cells typically confer genetic resistance against organ-specific autoimmunity by actively suppressing the effector functions of self-antigen-reactive T cells, thereby inducing peripheral tolerance. Although the overall number (frequency) of Tregs may be unchanged in MS patients compared to controls, studies indicate that their inhibitory function is impaired, which correlates with diminished immune control *in vivo* (Costantino et al., 2008).

Further studies have shown the absence of GSH in T cells does not interfere with TCR signaling but leads to a failure to reprogram cellular processes needed for robust clonal expansion and prevents the induction of aerobic glycolysis and the increase in cell size associated with T cell activation (Klein Geltink et al., 2017). In addition to GSH effects with T cells, a decrease in GSH plays similar roles in MS as it does in AD and PD, namely, to mark an increase in oxidative stress and inflammation in the respective disease (Choi et al., 2018). Current treatments such as dimethyl fumarate (DMF) are already approved to treat MS through stabilizing the transcription factor Nrf2 which then induces the expression of antioxidant response element genes such as GSH (Bresciani et al., 2023).

Overall, a decrease in GSH is seen across AD, PD, and MS. Its exact role in each disease differs slightly, but each revolves around GSH’s major role as an antioxidant. In AD, GSH depletion impacts microglia function and reduces β-amyloid clearance. In PD, GSH impacts the degeneration of dopaminergic neurons. Finally, in MS GSH loss impacts immune regulation and blood brain barrier integrity.

## 4 Immunopharmacological implications

Physiological levels of ROS, which are present in all living organisms, can activate both signaling pathways such as MAPK and ERK and transcription factors such as NF-κB (Sies and Jones, 2020; Dou et al., 2018; Bae et al., 2011; Huot et al., 1997). However, at non-physiologic levels ROS are highly toxic and cause widespread non-specific damage to many biological systems. GSH has long been implicated as a major antioxidant and redox regulator that functions to mitigate and alleviate this oxidative damage (Averill-Bates, 2023).

**FIGURE 3:**
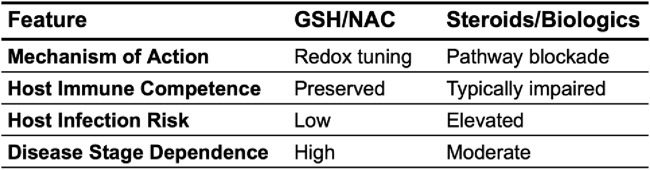
A direct comparison of glutathione and its precursors to typical immunosuppressive therapies.

However, the preceding sections of this paper establish that GSH is not merely a passive antioxidant, but an immune regulator and modulator with actions dependent upon the pathophysiologic context (Ghezzi, 2011) GSH acts to regulate the thresholds necessary for immunologic activation through redox-sensitive signal transduction pathways, influences antigen presentation and the development/differentiation of immune cells, and modulates the production of cytokines. As such, its actions as a signaling molecule may be even more critical than those of a ROS scavenger (Diotallevi et al., 2017). Because of its ability to assist in the regulation of oxidative stress, production of cytokines, and overall immune response, the use of GSH and precursors like NAC have become increasingly popular as potential medicinal agents for autoimmune and neurodegenerative diseases such as AD, PD, and MS that involve inflammation and unregulated immune response (Tieu et al., 2023; Kalamkar et al., 2022; Mandal et al., 2022; Smeyne and Smeyne, 2013). The value of GSH as an immunopharmacologic agent is that it can finetune immune response in a disease-dependent and redox state-dependent manner which differs from typical broad-effect immunosuppressive treatments (Diotallevi et al., 2017) (Figure 3).

**FIGURE 4:**
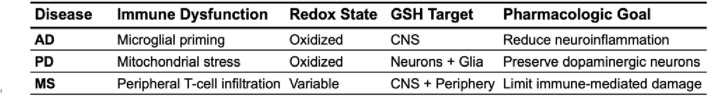
A comparison of redox dysregulation states and optimal glutathione targets in the disease states of Alzheimer’s, Parkinson’s, and Multiple Sclerosis.

The immunopharmacological effects of GSH are evident through several distinct but interconnected pathways, including regulation of inflammatory signaling networks like MAPK and NF-κB, buffering of pathologic ROS as well as maintenance of cellular ROS signaling, and modulation of both peripheral and central immune cell activity (Bae et al., 2011; Ghezzi, 2011; Limongi et al., 2019; Peterson et al., 1998). This means that the clinical effectiveness of GSH–and that of its precursors like NAC–is dependent not only on its capability to act as an antioxidant, but also the degree to which their pharmacodynamic properties are in line with the dysfunction in cellular signaling, redox imbalance, and immune response generated by specific disease states (Chai and Mieyal, 2023; Fraternale et al., 2021). However, these effects are contingent on the delivery of GSH or its precursors to the necessary targets (Vázquez-Meza et al., 2023). For states of systemic inflammation that involve cells of the peripheral immune system, while for neurodegenerative and neuroinflammatory diseases such as AD, PD, and MS, penetration of the CNS is necessary (Lin et al., 2024; Salem et al., 2015) (Figure 4).

This is complicated because as a polar, charged tripeptide, transport of GSH is significantly inhibited by the blood-brain barrier (BBB), thus limiting effective delivery to brain-resident immune cells such as microglia which are central drivers of neuroinflammatory pathology (Valdovinos-Flores and Gonsebatt, 2012; Aoyama, 2021). Multidrug resistance-associated proteins (MRPs) and most other cellular transporters of endogenous GSH are chiefly involved in mediating the efflux of GSH, not its uptake. This makes oral and parenteral administration of exogenous GSH problematic as it leads to poor accumulation in the CNS (Ronaldson and Davis, 2015). As previously discussed, neurodegenerative and autoimmune disorders such as AD, MS, and PD are associated with reduced GSH concentrations in the brain making supplemental GSH an attractive therapy if the limitations in delivery can be overcome (Smeyne and Smeyne, 2013).

As many treatments for neurological conditions have been hampered by limited permeability of the BBB, significant emphasis has been placed on research directed towards advanced drug delivery methods (Liu et al., 2025). The primary routes for permeation across the BBB–include passive diffusion (lipid soluble solutes), ABC transporter efflux, carrier-mediated influx, and receptor-mediated transcytosis (Abbott et al., 2010). Increasing CNS uptake of therapeutic agents has focused on increasing passive diffusion through liposomal encapsulation and transcytosis through conjugation with polymeric nanoparticles (Pandian et al., 2022; Zha et al., 2024; Hersh et al., 2022). While more research is needed on liposomal GSH uptake in the CNS, daily liposomal GSH supplementation has been shown to increase GSH levels in whole blood, erythrocytes, plasma and PMBCs within 2 weeks, showing increased passive diffusion of GSH across lipid membranes (Sinha et al., 2018).

More targeted CNS uptake has been attempted by conjugating polymeric nanoparticles with ligands like transferrin or lactoferrin (Zha et al., 2024; Teixeira et al., 2023). More efficient penetration has been shown through directly comparing GSH to a GSH monoethyl ester (GEE), showing that GEE significantly elevated intracellular GSH levels in mesencephalic cultures (Zeevalk et al., 2007). Improved nanocarrier technology targeting the CNS is a critical step towards treating disorders characterized by the coexistence of inflammation and oxidative stress where the immunologic properties of GSH show therapeutic benefit. While supplementation with exogenous GSH has shown therapeutic potential on its own, recent findings indicate advantages to combined treatment with other immunomodulatory or anti-infective agents (Vairetti et al., 2021).

As a combined medicinal treatment for viral infections such as COVID-19 or influenza, administration of GSH or NAC in conjunction with established anti-viral treatment has been found to improve lymphocyte response, reduce pro-inflammatory cytokines and reduce cellular oxidative injury (Tieu et al., 2023). Elevated intracellular GSH (or precursors like NAC) in autoimmune conditions has been shown to downregulate the activity of NF-κB which mediates pro-inflammatory gene transcription (Limongi et al., 2019). This has also shown potential benefit when used in combination with other immunotherapies because it results in a reduction of pathological inflammation without generalized suppression of the immune system or compromising the body’s adaptive immune response. These findings support adjunct GSH/NAC use as an immunotherapeutic in addition to well-established anti-viral and immunomodulatory treatments, leading to improved outcomes in conditions characterized by hindered immune control as a result of oxidative stress (Stanislaus et al., 2005; Wong et al., 2021; De Rosa et al., 2000).

As a pharmacologic therapeutic, optimizing the efficacy of GSH and minimizing its unintended effects means it is crucial to determine the correct timing, dose, and disease context (Hong et al., 2005). Treatment with exogenous GSH during acute infections or illnesses can amplify immune response by restoring optimum redox conditions for adequate immune cell signal transduction (Diotallevi et al., 2017). During the antigen recognition process, ROS are generated which function as second messengers to activate inflammatory signaling networks like MAPK and NF-κB, and GSH buffering maintains the conditions to support those pathways (Schieber and Chandel, 2014). Conversely, with conditions leading to neurodegeneration or chronic inflammation, oxidative stress persists long enough to lead to abnormal immune response pathways–particularly NF-kb–and prolonged treatment with GSH may lead to reduced redox tone and thus attenuating NF-kB, causing an anti-inflammatory response instead of an immuno-supportive one (Limongi et al., 2019; Tyuryaeva et al., 2023). Thus the dose, timing, and disease-specific inflammatory response are important pharmacological considerations with GSH treatment (Montero et al., 2023).

In addition to being a therapeutic target, oxidative stress has been shown to decrease the GSH:GSSG ratio which has proven useful as a clinically accessible, pharmacodynamic biomarker of immune redox tone for conditions such as malignancy (Zitka et al., 2012). Reduced GSH levels shown by decreases in this ratio–both in the plasma and intracellularly–reflect intracellular shifts in oxidative burden and are indicative of immune dysfunction and progression of various disease states such as infection, sepsis, and age-related immune system decline (Guerra et al., 2011; Morris et al., 2012; Keller et al., 1985). Further research indicates that GSH: GSSG is able to accurately distinguish viral and bacterial infections, offering critical clinical insight to contribute to more accurate diagnosis (Milhelm et al., 2025). As GSH: GSSG serves as an accurate representation of cellular redox status it is also valuable as a biomarker for patient classification, active monitoring of treatment and the patient’s physiologic response, and an easily obtained measure of immune exhaustion or oxidative burden (Rossi et al., 2006; Gomar et al., 2025; Sanchez-Rodriguez and Mendoza-Nunez, 2019; Giustarini et al., 2017).

GSH has also shown promise as a redox-based biomarker for neurodegenerative disease states. Recent advances in neuroimaging technology allowing for *in vivo* quantification of GSH levels in the brain have shown decreased GSH in patients with MS compared to healthy controls, with the degree of depletion correlating with disease severity and showing parallel longitudinal decline over time (Choi et al., 2018; Carvalho et al., 2014). Decreases in GSH levels commensurate with cognitive decline were observed in the hippocampi and frontal cortices of patients with AD, showing potential to be used as a clinical marker of disease staging and progression (Mandal et al., 2015). While a recent study demonstrated a lack of correlation between GSH levels in the putamen and body status in patients with PD, whole blood GSH was shown to be sensitive to aging with concentrations being correlated to severity of PD progression (Mischley et al., 2016).

## 5 Future directions

The clinical load of neurodegenerative diseases like AD and PD is projected to increase exponentially in coming years, with a commensurate increase in need for potential treatments. However, the immune response of the CNS is complex and involves a myriad of diverse cell populations such as astrocytes, microglia, macrophages, NK cells, and T and B cells (Aoyama, 2021). GSH continues to show promise as a therapeutic agent however its specific impact on individual types of immune cells remains poorly understood (Averill-Bates, 2023). It has been established that astrocytes are key to CNS metabolism of GSH, but further research is also required to explore the correlation between GSH levels in astrocytes and overall GSH content in the brain (Dringen and Arend, 2025).

Current GSH treatment for neurodegenerative disorders has been focused largely on trying to bypass the BBB (Liu et al., 2017). An area of growing interest involves the ability to not only enter the CNS but target specific immune cell types. A recent study demonstrated the ability of a dendrimer-conjugated NAC (D-NAC) to specifically target glial and astrocyte cells, indicating potential to alter GSH levels in specific cell populations without altering levels throughout the CNS (Nance et al., 2017; Turk et al., 2018). Future studies are warranted to explore more targeted GSH supplementation and its effect on treatment efficacy.

One of the most well-established properties of GSH is its role in maintaining redox homeostasis, but it is also emerging as a mediator of redox signaling through mechanisms such as “protein S-glutathionylation and/or GSNO-mediated protein S-nitrosylation” (Zhang and Forman, 2012). Further research is needed to elucidate the circumstances, specific molecular targets, other enzymes involved in the signaling process, and to map the involvement of GSH in redox signaling pathways (Aquilano et al., 2014).

An emerging area of research is that of immune memory in the brain (Neher and Cunningham, 2019). Redox mechanisms are critical to this process and help moderate defense signaling and response, and GSH is one of the key components of the redox signaling process (Morris et al., 2014). This redox signaling appears to be a critical component of immune system training and serves to preserve redox equilibrium. This in turn enables the “activation of defense signaling proteins and transcription factors” which helps to establish innate immune memory (Lindermayr and Yildirim, 2025). However, more evidence is needed to support this specific relationship along with further studies directed towards the effects of modulating levels of GSH on immune cell activity and programming in the CNS.

Because of the importance of GSH in many cellular and immunological processes and its potential as a biomarker, accurate measurement and analysis of GSH levels are critical to further research and analysis of its efficacy as a treatment. Traditional methods have typically included high performance liquid chromatographic assays and are characterized by underestimation of GSH and overestimation of GSSG due to the autooxidative properties of GSH (Nuhu et al., 2020). Newer measurement methods such as Magnetic Resonance Spectroscopy (MRS) and Positron Emission Tomography (PET) are less invasive and more cost-effective and show potential to be more accurate, but more research is needed to further validate and standardize results (Murray et al., 2024; Bottino et al., 2021; Qin et al., 2018).
